# Fatal rhino-orbito-cerebral mucormycosis infection associated with diabetic ketoacidosis post-COVID-19

**DOI:** 10.1590/0037-8682-0358-2021

**Published:** 2021-07-12

**Authors:** Paula Bonates, Guilherme Augusto Pivoto João, Kátia Santana Cruz, Marcelo de Souza Ferreira, Djane Clarys Baía-da-Silva, Maria Eduarda Leão de Farias, José Diego Brito-Sousa, Monique Freire Santana, Luciana Aires de Oliveira, Ana Cláudia Alves Cortez, João Vicente Braga Souza, Marcus Vinicius Guimarães Lacerda

**Affiliations:** 1 Fundação de Medicina Tropical Dr. Heitor Vieira Dourado, Manaus, AM, Brasil.; 2 Hospital e Pronto Socorro Dr. João Lúcio Pereira Machado, Manaus, AM, Brasil.; 3 Universidade do Estado do Amazonas, Manaus, AM, Brasil.; 4 Fundação Oswaldo Cruz, Instituto Leônidas & Maria Deane, Manaus, AM, Brasil.; 5 Fundação Centro de Controle de Oncologia do Estado do Amazonas, Manaus, AM, Brasil.; 6 Instituto Nacional de Pesquisas da Amazônia, Manaus, AM, Brasil.

**Dear Editor,**

With the progression of the COVID-19 pandemic, the use of corticosteroids and other immunosuppressive drugs to manage critically ill patients has been widely disseminated and likely contributed to the increase in secondary infections and uncontrolled glycemic status^1^ ^,^ ^2^. The SARS-CoV2 virus can directly infect T cells and make patients susceptible to secondary infections and severe COVID-19 infection^3^. Among fungal infections, cases of invasive pulmonary aspergillosis, invasive candidiasis, and pneumocystis have been described^2^ ^-^ ^4^. Here we report a fatal case of rhino-orbito-cerebral mucormycosis in a patient with uncontrolled diabetes after a COVID-19 infection.

**FIGURE 1:**
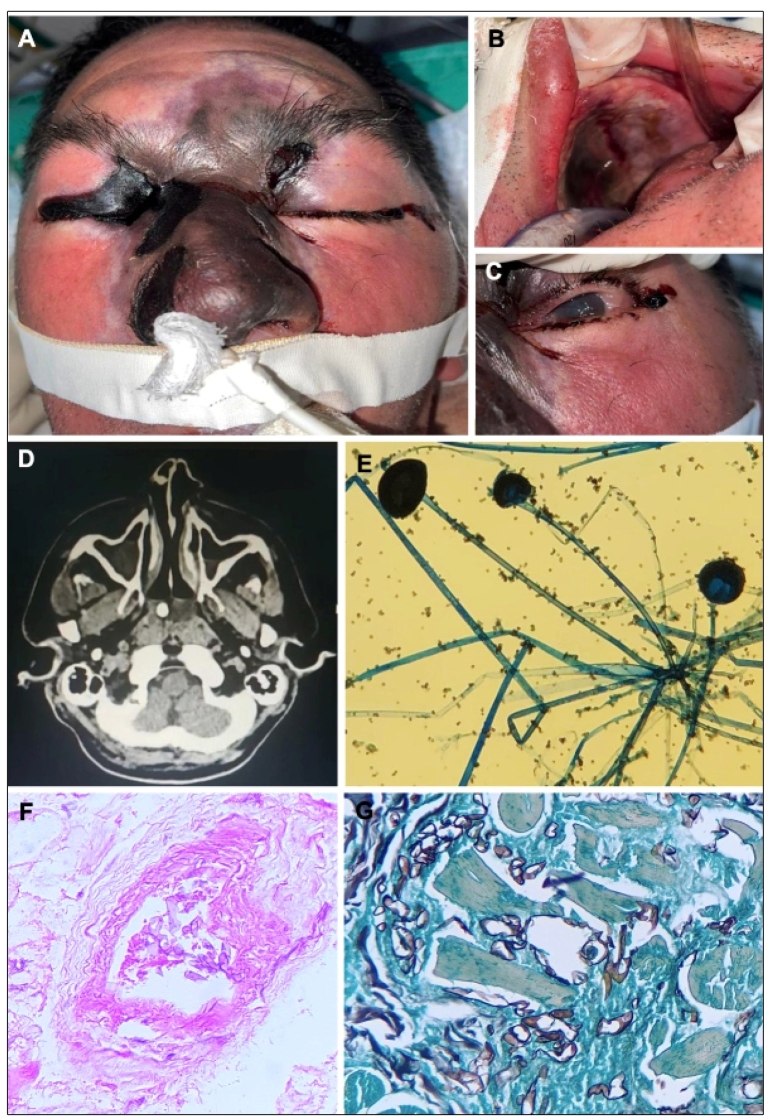
Clinical and histopathological aspects of fatal rhino-orbito-cerebral mucormycosis. Frontal view of the face showing areas of extensive necrosis in the nose, both orbits **(A)**, and palate **(B)**. Pupillary opacity **(C)**. Computed tomography of the sinuses showed infiltration of the maxillary and ethmoid sinuses **(D)**. Direct examination of samples collected with wide non-septate hyphae with an angle of 90 degrees **(D)**. After fast growth of beige colonies in tissue culture, a microscopic examination showed rhizoids at a base of sporangia **(E)**. Histology showing wide paucisepte hyphae (5-15 microns in diameter) **(F and G)** in the intravascular space with necrosis of the adipose tissue **(F)** (F: hematoxylin and eosin, 400×; G: Grocott-Gomori, 400×).

A 56-year-old male resident of Manaus-Brazil with insulin-dependent type 2 diabetes mellitus was admitted to the emergency department of a tertiary health care with an altered blood glucose level and an infection in the right eye. Diabetic ketoacidosis was confirmed. The patient required orotracheal intubation and admission to the intensive care unit. The patient tested immunoglobulin G-positive for SARS-CoV-2 infection upon admission. Two weeks prior, the patient had flu-like symptoms and dyspnea. On physical examination, the patient presented with areas of extensive necrosis in the nose, orbits (Figure 1A), and palate (Figure 1B). Computed tomography of the sinuses showed infiltration of the maxillary and ethmoid sinuses without drainage of the secretion (Figure 1D). The next day, the lesions worsened and started to expand to the soft palate. Pupillary opacity was observed (Figure 1C). A microscopic examination showed wide hyphae with a 90-degree angle detected by direct sample examination after growth in tissue culture using Sabouraud dextrose agar (rhizoids at the base of sporangia) (Figures 1E, 1F). Histopathological findings from the biopsy of the maxillary sinus included aseptate hyphae in necrotic tissue (Figures 1G, 1H). Despite receiving antifungal treatment with amphotericin B under medical care in the hospital, the patient died on the fourth day of hospitalization. 

**FIGURE 2:**
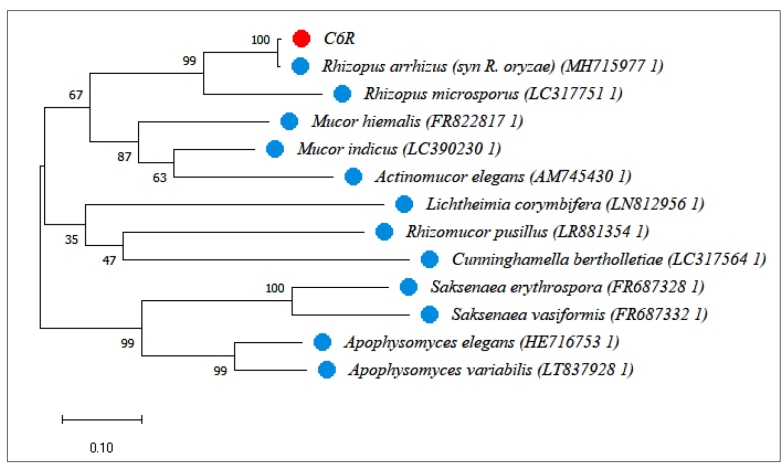
Phylogenetic tree constructed using the ITS1-5.8S rDNA-ITS2 sequences. Sequences are labeled with their database accession numbers. Support values are from Bayesian inference, and maximum likelihood analyses (values above and below the branches, respectively) were conducted in MEGA X software (Molecular Evolutionary Genetics Analysis - https://www.megasoftware.net/). The red dots indicate the isolate from this study, while the blue dots indicate the main species causing mucormycosis in Latin America.

Colony (fluffy and gray-white) obtained from tissue culture on potato dextrose agar plus cloranfenicol at 30°C for two days was subjected to manual DNA extraction. The internal transcribed spacer 1 (ITS1) and ITS2 regions and 5.8S ribosomal DNA were amplified by polymerase chain reaction using the universal primers ITS1 and ITS4, as described previously^5^. Sequencing was performed using the BigDye® Terminator v3.1 Cycle Sequencing (Applied Biosystems) and the above primers on an individual basis. The sequence was manually edited using Sequencher 5.4.6 software. As a result, the sequence showed 99% similarity with the sequence of *Rhizopus arrhizus* (syn. *Rhizopus oryzae*) (access number MH715977.1) deposited in NCBI BLAST. A phylogenetic tree was constructed using MEGA v.10.0.2 software to verify the genetic and evolutionary relationships among the current study sequence and those of the main species causing mucormycosis in Latin America (Figure 2). The sequence extracted from the current study *R. arrhizus* (syn *R. oryzae*) has been deposited in GenBank under accession number MZ344145.

Rhino-orbito-cerebral mucormycosis is a rare acute opportunistic fungal infection with a rapid and lethal progression that affects the nose and paranasal sinuses of the head and neck^6^. *R. arrhizus* is the most commonly isolated agent worldwide^7^. Mucormycosis is a critical threat to patients with uncontrolled diabetes or other predisposing systemic conditions such as corticosteroid use^6^. SARS-CoV-2 infection may favor immunosuppression, which, when associated with glycemic dysregulation, favors the onset of a severe fungal infection that results in rhino-orbito-cerebral mucormycosis. To the best of our knowledge, this is the first description of rhino-orbito-cerebral mucormycosis after a SARS-CoV-2 infection with a fatal outcome in the Americas. This emphasizes the need for disease surveillance in the COVID-19 scenario. Health professionals must act immediately in cases of suspected mucormycosis infection, especially in patients with uncontrolled diabetes who take corticosteroids or previous exposure to antifungal drugs that lack activity against Mucorales^8^. Its early diagnosis and treatment are of paramount importance in preventing its progression, especially because of its angioinvasive characteristics^9^.
